# Genome Sequence of Bacillus velezensis SGAir0473, Isolated from Tropical Air Collected in Singapore

**DOI:** 10.1128/genomeA.00642-18

**Published:** 2018-07-05

**Authors:** Serene B. Y. Lim, Ana Carolina M. Junqueira, Akira Uchida, Rikky W. Purbojati, James N. I. Houghton, Caroline Chénard, Anthony Wong, Sandra Kolundžija, Megan E. Clare, Kavita K. Kushwaha, Deepa Panicker, Alexander Putra, Nicolas E. Gaultier, Balakrishnan N. V. Premkrishnan, Cassie E. Heinle, Vineeth Kodengil Vettath, Daniela I. Drautz-Moses, Stephan C. Schuster

**Affiliations:** a Singapore Centre for Environmental Life Sciences Engineering, Nanyang Technological University, Singapore; b Departamento de Genética, Instituto de Biologia, Universidade Federal do Rio de Janeiro, Rio de Janeiro, Brazil; c Asian School of the Environment, Nanyang Technological University, Singapore

## Abstract

Bacillus velezensis strain SGAir0473 (*Firmicutes*) was isolated from tropical air collected in Singapore. Its genome was assembled using short reads and single-molecule real-time sequencing and comprises one chromosome with 4.18 Mb. The genome consists of 3,937 protein-coding genes, 86 tRNAs, and 27 rRNAs.

## Abstract (precis)

Bacillus velezensis strain SGAir0473 (*Firmicutes*) was isolated from tropical air collected in Singapore. Its genome was assembled using short reads and single-molecule real-time sequencing and comprises one chromosome with 4.18 Mb.

## GENOME ANNOUNCEMENT

Bacillus velezensis is a Gram-positive rod-shaped aerobic bacterium classified in the phylum *Firmicutes*. This bacterium was first isolated from a brackish water sample collected from the river Vélez in Spain (1). It was isolated from an extremely high-salt environment (12% [wt/vol]) utilizing a test that screens for surfactant-producing bacteria (1). Since then, B. velezensis has been found in diverse habitats, including cotton waste (2), wheat anthers (3, 4), and soil (5). Past studies have also highlighted the ability of B. velezensis to produce antimicrobial metabolites (6) and antibiotics (7) and have also demonstrated its involvement in a wide spectrum of antifungal activities (8).

B. velezensis strain SGAir0473 was isolated from air in an outdoor seaside location in Singapore (global position system coordinates 1.391°N, 103.992°E). Air was drawn and directly impacted onto brain heart infusion agar (Becton, Dickinson, USA) using the Andersen single-stage impactor (SKC, USA). After initial incubation at 30°C, subsequent colony isolation was carried out by culturing on Trypticase soy agar at 30°C. Finally, the pure culture was grown in Luria-Bertani broth overnight before DNA extraction.

Extraction of genomic DNA was performed using the Wizard genomic DNA purification kit (Promega, USA) according to the manufacturer’s standard protocol. After extraction, library preparation was performed with the SMRTbell template prep kit 1.0 (Pacific Biosciences), followed by single-molecule real-time (SMRT) sequencing on the PacBio RS II (Pacific Biosciences) platform. Short reads were also generated with a MiSeq (Illumina) 300-bp paired-end run using whole-genome shotgun libraries constructed with the TruSeq Nano DNA library preparation kit (Illumina).

*De novo* assembly of the 26,993 long subreads generated on the PacBio RS II platform was accomplished using Hierarchical Genome Assembly Process (HGAP, version 3), which is included in the PacBio SMRT Analysis 2.3.0 package (9). The assembly was then polished and error corrected with the 770,556 MiSeq reads using Quiver (9) and Pilon version 1.16 (10). The final assembly generated one chromosomal contig with a total size of 4,184,178 bp (38.26-fold coverage) and a G+C content of 45.96% evaluated using QUAST (11). The genome was unable to be circularized using Circlator (12). Average nucleotide identity (ANI) analysis, performed with MiSI (13), indicated a 99.26% match to B. velezensis.

Genome annotation was performed with NCBI’s Prokaryotic Genome Annotation Pipeline (PGAP) version 4.2 (14). A total of 4,198 genes were predicted, including 3,937 protein-coding genes, 9 copies each of 5S, 16S, and 23S rRNA genes, 86 tRNA genes, 5 noncoding RNA genes, and 143 pseudogenes.

Functional annotation with Rapid Annotations using Subsystems Technology (RAST) (15–17) highlighted genes that were associated with osmotic stress response (14 genes) and alkanesulfonate metabolism (8 genes), which could explain how this bacterium survives in high-salt (18) or surfactant-rich (19, 20) environments. Genes related to sporulation and dormancy (116 genes) were also found. This indicates that the species might be capable of surviving in other extreme environments, such as air.

### Accession number(s).

The genome sequence of Bacillus velezensis strain SGAir0473 has been deposited in DDBJ/EMBL/GenBank under the accession number CP027868.
